# Secondary Metabolites in *Xylella fastidiosa*–Plant Interaction

**DOI:** 10.3390/pathogens9090675

**Published:** 2020-08-20

**Authors:** Marzia Vergine, Francesca Nicolì, Erika Sabella, Alessio Aprile, Luigi De Bellis, Andrea Luvisi

**Affiliations:** Department of Biological and Environmental Sciences and Technologies, University of Salento, 73100 Lecce, Italy; marzia.vergine@unisalento.it (M.V.); erika.sabella@unisalento.it (E.S.); alessio.aprile@unisalento.it (A.A.); luigi.debellis@unisalento.it (L.D.B.); andrea.luvisi@unisalento.it (A.L.)

**Keywords:** Secondary metabolites, *Xylella fastidiosa*, plant-pathogens interaction, plant disease, plant defense

## Abstract

During their evolutionary history, plants have evolved the ability to synthesize and accumulate small molecules known as secondary metabolites. These compounds are not essential in the primary cell functions but play a significant role in the plants’ adaptation to environmental changes and in overcoming stress. Their high concentrations may contribute to the resistance of the plants to the bacterium *Xylella fastidiosa*, which has recently re-emerged as a plant pathogen of global importance. Although it is established in several areas globally and is considered one of the most dangerous plant pathogens, no cure has been developed due to the lack of effective bactericides and the difficulties in accessing the xylem vessels where the pathogen grows and produces cell aggregates and biofilm. This review highlights the role of secondary metabolites in the defense of the main economic hosts of *X. fastidiosa* and identifies how knowledge about biosynthetic pathways could improve our understanding of disease resistance. In addition, current developments in non-invasive techniques and strategies of combining molecular and physiological techniques are examined, in an attempt to identify new metabolic engineering options for plant defense.

## 1. Introduction

Plants have evolved the capability to synthesize and accumulate a myriad of structurally diversified small molecules known as secondary metabolites. An important role of secondary metabolites is the chemical defense against pathogens. The compounds are stored in specialized cells or tissues (e.g., trichomes or epidermal cells) and are referred to as phytoanticipins, which are produced independently from the presence of a pathogen, or phytoalexins, which are synthesized de novo after a pathogen attack [1,2,3]. These compounds have a protective function against microbial pathogens, viruses, herbivores (mainly insects) and other plants [3,4]. Some secondary plant metabolites also have significant medicinal value, therefore knowledge of the relevant biosynthetic pathways and the stimuli that determine their production can be applied in plant cell cultures and for the metabolic engineering of plant cells [5].

Plant secondary metabolites are commonly classified into three main groups based on their chemical characteristics and metabolic origins: terpenes (such as carotenoids and plant volatiles), phenolic compounds (such as tannins, coumarins, lignin, and flavonoids), nitrogen/sulphur-containing compounds (such as alkaloids, cyanogenic glucosides, and non-protein amino acids/glucosinolate) [6,7].

Thus, the pathways of secondary metabolites are very complex and sometimes interact with each other. Despite this high degree of complexity, the importance of secondary metabolites in the plants’ processes of defense against microorganisms is well known [4]. Classical examples of phytoanticipins are avenacin and α-tomatine, two saponins (amphipathic triterpenoid or steroid glucosides) found in oats and tomato, respectively. Avenacin protects oats from the pathogen *Gaeumannomyces graminis* var. *tritici*, which can colonize other cereals, such as barley and wheat, that do not produce this saponin, and the ability to inactivate α-tomatine through the secretion of a tomatinase is crucial for the pathogenicity of *Septoria lycopersici* [3,4].

Of the phytoalexins involved in disease resistance, the most investigated are camalexin (an indole alkaloid) in *Arabidopsis* [4,8] and pisatin (isoflavonoid), one of the first compounds purified and chemically characterized in *Pisum* spp. [9,10]. However, different biotic agents have been found to selectively elicit the synthesis of various phytoalexins at different levels in peanut plants [11].

The activation of the defense response in plant tissues and organs is mediated by signals like ethylene or salicylic acid (involved in systemic acquired resistance (SAR)), which require the synthesis of other signal molecules. Compounds such as azelaic acid (a catabolite of free unsaturated fatty acids) and pipecolic acid (a lysine degradation product) may help plants to respond more quickly to further attacks by pathogens [12]. These most recent findings have focused attention onto the pathogenesis mechanisms of very harmful pathogens such as *Xylella fastidiosa* (*Xf*). This xylem-limited Gram-negative bacterium causes severe diseases in several crops and leads to significant economic losses worldwide [13,14,15]. Studies on the *Xf*–plant interaction have led to the identification of several small signal molecules produced by the plant in response to pathogenic attacks and involved in active defense mechanisms that can also be used as infection biomarkers [16,17,18].

## 2. New Insights on Plant Response to *X. fastidiosa*

Over 500 plant species are susceptible to *X. fastidiosa* infection [19]. Although many host plants are asymptomatic [20], the typical symptoms include leaf-scorch and wilting and ultimately the death of infected plants results in most cases, mainly due to interference with xylem vessel performance and sap flow [21,22]. In the United States, Brazil, and Costa Rica, this xylem-limited plant pathogenic bacterium is associated with several diseases such as Pierce’s disease (PD) in *Vitis* spp. [23], citrus variegated chlorosis (CVC) in *Citrus* spp. [24], and coffee leaf scorch in *Coffea arabica* [25]. *Xf* is already widely distributed in the Americas and has been detected in Asian countries such as Iran and Israel in *Prunus dulcis* (almond leaf scorch (ALS)) [26,27] and Taiwan in *Vitis vinifera* (PD) [28], and has been known to be present in Europe since 2013 after its official detection in Apulia (southern Italy). Here, it mainly affects *Olea europaea* (as Olive Quick Decline Syndrome (OQDS)) [29]. The recent identification of all three main subspecies of *Xf* (*fastidiosa*, *multiplex*, and *pauca*) in Europe (e.g., in Italy, France, and Spain) extends the threat to several other crops, including almond, citrus, and grapevines, but also ornamental trees, elms, oaks, oleander and sycamores [30,31].

This pathogen is therefore well established in several areas, and the management of diseases caused by *Xf* is to date based on the use of pathogen-free propagation plant material, quarantine measures, uprooting of infected plants, and vector control [22]. No cure of the diseases as been found due to the lack of effective bactericides and the difficulties in accessing xylem vessels, where the pathogen establishes and produces cell aggregates and biofilm [32,33,34]. Numerous studies have been conducted with the aim of limiting the occurrence and spread of *Xf*, due to this absence of any effective treatment. To date, most research programs have focused on understanding the factors and mechanisms of resistance observed in host plants cultivars. Citrus plants and grapevines affected by *Xf* have also been investigated, to establish how the endophytic bacterial community can interact with pathogen growth through competition for space and nutrients, the excretion of lytic enzymes or the production of antibiotics, and how interference with pathogen signaling and the degradation of pathogen toxins or virulence factors can result in a lower bacterial population and reduced symptoms [35,36]. Olive microbiome research has also indicated that mild symptomatic trees of the cv FS17 were dominated by fungi, while the fungi/bacteria ratio was inverted in trees of the susceptible cv Kalamata, suggesting that some endophytes may have antagonistic activity towards *Xf* [37]. In addition, maintaining healthy microbiota and the presence of cultivar-specific microbes may support the resilience of the resistant olive cultivar Leccino to *Xf* infection [38]. This evidence was also confirmed in other pathosystems where experimental evidence suggests that plant colonization by specific bacterial endophytes is marked by a change in the expression of key genes in central metabolic and by priming expression of innate disease resistance pathways in plants that result in the reduction of disease [39,40]. At the same time, endophytic fungi can influence the plant’s metabolism by increasing the content of antioxidant compounds in the host [41].

More findings about ionomic differences have emerged, and it has been observed that higher concentrations of calcium (Ca) and manganese (Mn) levels may contribute to protecting against disease caused by *Xf* infection, as observed previously in the responses of tobacco, grapes, pecans, and blueberries [42,43,44,45]. Bacterial growth, aggregation, and biofilm formation may be affected by xylem sap components [46] and by lipid profile [47], and thus several studies have focused on analyzing xylem-localized small molecules that can potentially inhibit the growth and movement of *Xf*. Promising in this area was the study conducted on the role of different classes of lipids in the *Xf pauca*–*O. europaea* interaction in the modulation of biofilm, their possible use as defense plant signals and finally, as new targets for the development of treatments for OQDS [47]. In grapevine a transient increased production of phenolic compounds was observed following *Xf* infection, as long as the hosts have the resources to support the production of these defense-associated secondary metabolites [48]. The content of catechin (a phenolic compound), digalloylquinic acid (a polyphenolic compound), and astringin (a glycoside) may increase in the xylem sap, and multiple catechins, procyanidins (flavonoids), and stilbenoids (phenolics) were found at higher levels in xylem tissues in response to *Xf* infection [48,49]. For example, cathechin in infected grapevine was quantified in higher concentration (131.5 µg mL^−1^) than that (116 µg mL^−1^) tested in vitro with anti-*Xf* activity effect [45]. In citrus, the hesperidin has been associated with lesions caused by *Xf* [50], and it is probably involved in natural defense or resistance mechanisms against *Xf* in sweet orange varieties. Thus, of the proposed defense mechanisms, differences in the quality–quantitative profile of secondary metabolites may represent a more successful attempt by the plant to cope with pathogen invasion.

## 3. Profiling Change of Metabolites in Economic Plants Affected by *X. fastidiosa*

Considering the extraordinary structural complexity of secondary plant metabolites and their possible role in the defensive responses of each species, we discuss here the compound classes for which chemical, biochemical, and genetic studies that indicate infection-limiting functions in the plants affected by *Xf*. Moreover, we summarize in Table 1 the secondary metabolites found in vivo in plants associated with *Xf* infection.

### 3.1. Citrus spp.

*Xf* subsp. *pauca* has caused critical economic losses in the Brazilian citrus industry. This disease, known as CVC, was first detected in the mid-1980s [51], and the pathogen can infect every cultivar of *Citrus* species and hybrids. Still, the severity of the symptoms may change depending on the host genotype. In most cases, CVC does not cause death, but infected trees always display a reduced vigor and growth rate. On the leaves, chlorotic lesions appear on the upper side. While, inside the leaf, the fastidian gum appears, an exopolysaccharide associated with the formation of biofilms inside the xylem vessels probably involved in bacterial pathogenicity [52]. Affected fruits are smaller, harsher, and ripen earlier, with lesser juice content and higher acidity [53]. To find a potential cure against this economically significant disease, research aimed to understand the resistance mechanisms has been conducted. De Souza et al. [54,55] constructed two expressed sequence tags (EST) libraries from sweet orange, *Citrus sinensis* (L.) Osbeck, with or without CVC symptoms. Using an in silico hybridization strategy, the authors found 37 genes having significant up- or down-regulation. The down-regulated transcripts were associated with metabolism, protein modification, energy, and transport facilitation, while the up-regulated transcripts were related to metabolism, defense response, and adaptation to stress conditions. Specifically, the study showed an up-regulation of transcripts representing genes involved with oxidative stress were in plants with CVC, such as peroxidases and copper/zinc superoxide dismutase. In response to xylem vessels blocked by bacterial biofilm, the affected tree may modulate the level of superoxide radicals (O_2_^−^), triggering Superoxide dismutase to produce hydrogen peroxide, which can further react with the phenol group of monolignol, in the presence of peroxidase, to produce lignin components with strength and defense function such as also observed by Sabella et al. [56] in putative tolerance of olive tree to *Xf*. A defense response model induced by *Xf* was proposed by de Souza et al. [57] in which one month from inoculation with *Xf*, cell wall degradation products, such as lipopolysaccharides, extracellular polysaccharides and adhesins, could function as non-specific elicitors and trigger a basal resistance response, which in the end leads to defense responses, with a signaling role for salicylic acid, methyl salicylic acid, ethylene, and jasmonic acid. Two months from inoculation, other genes involved in resistance are activated that interfere with the multiplication of the bacterium in the plant, limiting the symptoms and the disease.

Also, the accumulation of phenolic compounds was reported as a response to *Xf* in CVC-resistant citrus (*Citrus reticulata*) with the induction of phenylpropanoid and flavonoid biosynthesis genes taking place within 24 h after inoculation in “Ponkan” mandarin (*Citrus reticulata* Blanco), a CVC-resistant citrus variety [58].

Alves et al. [59] have studied how *Xf* colonizes and spreads within xylem vessels of sweet orange *Citrus sinensis* cv Pêra. Initially, *Xf* attaches to the cell wall, then follows an increase in the number of bacteria, the production of strand-like material, and the formation of biofilm. In xylem vessels of *C. sinensis* infected by *Xf*, hesperidin, produced inside leaf petioles by citrus plants, was also often present, but it was not observed in healthy plants [60]. To understand the correlation between the ability to accumulate hesperidin and the possible tolerance to CVC bacterium, Soares et al. [61] developed a rapid and sensitive high performance liquid chromatography (HPLC) method for the quantitative determination of hesperidin. Later on, the authors showed as, in Brazilian *C. sinensis* grafted on *C. limonia* cv. Pêra, the rootstocks interfere in the metabolism of the scion, determining a higher concentration of flavonoids (hesperidin) in leaves of symptomatic CVC-infected plants compared to asymptomatic and control plants, suggesting that the increase of this flavonoid can hence reduce the susceptibility of sweet orange to this bacterium [62].

### 3.2. Olea europaea L.

Olive trees infected by *Xf* subsp. *pauca* were reported in Argentina [63,64], Spain [65,66], and Brazil [67], and also infected by *Xf* subsp. *multiplex* was reported in California [20,68] and in both cases showed limited symptoms. Conversely, *Xf* subsp. *pauca* strain “De Donno” was identified in olive trees for the first time in Italy (Apulia region) in 2013 [29] associated with Olive Quick Decline Syndrome (OQDS), destroying not only local olive oil production, but also the typical landscape characterized by monumental olive trees [69,70]. To date, the mechanism of infection and the plant’s defensive response to pathogen attack and colonization, in this species, are still not well understood. Most of the considerations on the defense mechanisms in the olive tree to the pathogen *Xf* are based on the different susceptibility to infection of the olive cultivars present in Salento area (southern Apulia), where olive represents the principal crop. In the infected area, the cultivars Cellina di Nardò and Ogliarola salentina are the most cultivated. These two cultivars are characterized by high susceptibility to the pathogen and, due to high incidence of infection and severity of the symptoms, these olive trees very often face desiccation and death. Conversely, few are the other cultivar, such as Leccino, considered resistant to *Xf*, due to the low bacterial titer in the plant tissues [71,72,73,74]. Studies have indicated that infected field tree or potted inoculated plants of cultivar Leccino have a lower bacterial titer (4 × 10^4^ CFU∙mL^−1^) than Ogliarola salentina or Cellina di Nardò (2 × 10^6^ CFU∙mL^−1^) [15], underlying the resistant behavior of this cultivar. Thus, most Leccino plants are not just characterized by lower disease severity compared to Ogliarola salentina or Cellina di Nardò, a behavior commonly associated to tolerance which is defined as the host ability to reduce the effect of the pathogen infection. The lower bacteria titer observed in inoculated Leccino plants or naturally infected ones suggest the presence of resistance traits, which are those that reduce the extent of the pathogen infection [75]. Luvisi et al. [76] analyzed the phenolic composition of olive cultivars (susceptible and resistant) in response to *Xf* infection. Significant findings provide differences in some minor phenolic compounds but not in those commonly associated with resistance mechanisms activated against other pathogens in olive trees. In detail, a significant reduction of hydroxytyrosol glucoside (a phenolic compound) was observed in naturally infected plants, although lower in the cultivars resistant to *Xf*, and a significant increase in the amount of quinic acid—cyclic polyols are a key intermediate in the biosynthesis of chlorogenic acids, which are synthesized in planta by esterification of phenolic compounds, trans-hydroxycinnamic acids, with quinic acid—in both cultivars. Similar results on quinic acid have also been reported by Wallis et al. [49] in a study conducted on grapes with the symptoms of PD, suggesting a possible link between the behavior of olive tree and grape infected with *Xf* and the possible use of such compound as a marker of infection. Results were confirmed in a study by Sabella et al. [56], where the analysis of the phenolic profiles of two cultivars (Cellina di Nardò and Leccino) highlighted a reduction of the glucoside hydroxytyrosol and, only in Leccino, an increase in quinic acid and a significant increase in lignin compared to the sensitive cultivar. In this frame, a higher level of quinic acid in naturally infected susceptible cultivars Ogliarola and Cellina di Nardò was further confirmed by the research conducted by Girelli et al. [18], thus further supporting the possible use of this molecule as a biomarker for the disease.

Novelli et al. [17] evaluated the potential defense role of the secondary metabolites in olive plants exposed to *Xf* and reported the differences in the total content of simple phenols, flavonoids, and tannins between Cellina di Nardò and Leccino. In infected Leccino plants, higher amounts of flavonoids (such as quercetin, kaempferol and genistein), tannins, oleanolic acid (a triterpenoid), kynurenic acid (a tryptophan metabolite), and the phenolic signal molecule salicylic acid was observed compared to Cellina di Nardò plants. The higher levels of these compounds only in infected Leccino samples indicate their possible involvement in defense response to *Xf* infection.

### 3.3. Prunus Dulcis (Mill.) D.A. Webb

ALS was identified for the first time in California in the 1950s, and the disease was well known as the “Golden Death” because of its characteristic leaf yellowing and scorching symptoms. The main symptoms are leaf scorching (beginning at leaf margins), with a yellow or orange halo preceding the drying, a necrotic leaf margin, a significant tree decline over 3–8 years, and, of course, decreased almond yields [77,78]. Two genetically different *Xf* strains have been found in almond, *Xf* subspecies *multiplex* and *fastidiosa* including one almond group that can also cause PD [79], and recently in Europe *Xf* subsp *pauca* (ST53) [80] and *multiplex* (ST6) have also been isolated [81]. There is currently very little information about ALS, how the disease affects the tree, and how it could potentially be managed, compared to other diseases caused by *Xf.*

Most of the studies conducted on *Prunus* spp. have mainly focused on the use of different rootstocks and the related effects of tolerance/susceptibility to *Xf* on the scion [82,83]; as already observed in grapevine [49], the aim was to identify rootstocks with the capacity to reduce symptom progression, to influence plant vigor, *Xf* titer, and xylem sap phenolic levels. The research conducted by Wilhelm et al. [84], moreover, has attempted to correlate the already known different susceptibilities of various cultivars to ALS with the different composition of the xylem sap. The results showed that *Xf*-resistant cultivars (Butte and Carmel) tended to have higher concentrations of total phenolic compounds compared with susceptible cultivars Peerless and Sonora, suggesting a possible role of phenolic compounds in *Xf* resistance [84].

### 3.4. Vitis spp.

*Xf* subsp. *fastidiosa* was first associated with PD of grapevine (*Vitis vinifera*) in 1880 [85,86]. Since that moment, PD has been economically prominent, particularly in the south-western USA where it is endemic and is the principal factor limiting the development of a grape industry based on the high-quality *V. vinifera* and *V. labrusca* grapes [87]. PD has destroyed more than 35,000 acres of grapevine in southern California [88], menacing, de facto, the country’s $30 billion wine industry [89]. In the case of PD, researchers have tried to identify and evaluate various natural, antibacterial substances against *Xf*.

By examining the induction of phenolic compounds in “Thompson Seedless” grapevines inoculated in vivo with *Xf*, Wallis and Chen [48] observed (after two months post-inoculation) the increased levels of catechin, digalloylquinic acid, and astringin in xylem sap, as well as multiple catechins, procyanidins, and stilbenoids in xylem tissues. Moreover, precursors to lignin and condensed tannins in xylem cell walls also increased. However, six months after the inoculation, plants had significantly reduced phenolic levels in xylem sap and tissues, suggesting that even though grapevine can initially respond to infections with a high production of phenolic compounds, subsequently PD causes a decline in host plants, reducing their efficiency in the synthesis of phenolic compounds associated in PD defense.

In an afterward study, Wallis et al. [49] focused on the effect of rootstocks on PD symptom progression, *Xf* growth, and levels of defense-associated phenolic compounds in two different grapevine cultivars. Six months post-inoculation, caftaric acid was, along with the defense-associated hormone methyl salicylate and quinic acid, significantly higher in infected grapevines compared to plants that were not infected. This result suggests a connection between the defense response to *Xf* infection mediated by caftaric acid and the higher production of quinic acid with consequent effects on symptoms of PD and *Xf* titers. Therefore, it is plausible that variances in constitutive levels of phenolic compounds could vary among cultivars, resulting in differences in PD symptom development [90].

Finally, the study of Zaini et al. [91] about the molecular profiling of *V. vinifera* affected by *Xf* shows metabolites with known antimicrobial and signaling functions strongly influenced by disease onset. In particular, the authors observed the up-regulation of sequences coding for chalcone and stilbene synthases, the increased abundance of transcripts coding for terpene synthases, and a further increase of transcripts related to the synthesis of compounds such as erythritol and 2-deoxyerythritol, 1,2-anhydro-myoinositol and arbutin; the latter are known to inhibit pathogen growth and biofilm formation.

**Table 1:** Main secondary metabolites identified through in vivo analysis of economic plants (*Citrus* spp., *Olea europaea*, and *Prunus dulcis* and *Vitis* spp.) affected by *Xylella fastidiosa*.

Plant	Evidences	References
*Citrus spp.*	Induction of genes involved in phenylpropanoid and flavonoid biosynthesis	[54,55,57,58]
	Presence of hesperidin in areas where tissues disrupted by *X. fastidiosa*	[59]
	Induction of hesperidin production	[61]
	Increase of flavonoids in leaves and coumarins in roots	[62]
*Olea europaea* L.	Reduction of hydroxytyrosol glucoside and increase of quinic acid	[76]
	Reduction of hydroxytyrosol glucoside and increase of quinic acid and lignin content	[56]
	Higher content of quinic acid in infected leaves	[18]
	Increase of flavonoids (such as quercetin, kaempferol and genistein), tannins, oleanolic acid, salycilic, and kynurenic acids	[17]
*Prunus dulcis* (Mill.) D.A. Webb	Higher concentrations of total phenolic compounds in resistant than susceptible cultivars	[84]
*Vitis spp.*	Increase of catechin, digalloylquinic acid, astringin, multiple catechins, procyanidins, stilbenoids, lignin and condensed tannins	[48]
	Increase caftaric acid, methyl salicylate and quinic acid	[49,90]
	Increase of transcripts for terpene, chalcone, stylbene synthases, erythritol and 2-deoxyerythritol, 1,2-anhydro-myo-inositol, arbutin; glycosidase and tyrosinase	[91]

## 4. Conclusions

Alternative secondary metabolite compounds in plants can be extremely valuable in terms of crop protection, particularly with increasing concerns about the use of synthetic pesticides and shifts in pest management strategies, as recent EU legislation illustrates [92]. Today, phytochemicals are used to develop new commercial applications against several crop pathogen agents [93]. This issue is particularly pressing given the rapid and extensive spread of plant pests worldwide, and because many years of crop selection for yield or palatability traits has significantly reduced phenotypic and genetic diversity, leading to a loss of resistance [94]. In addition, the increase in the international plant trade has led to the introduction of new pathogens and related diseases, and thus substantial economic losses. This is evident in Italy with the OQDS in the olive groves, which represents probably the largest-scale destruction of trees due to pathogens in the last 50 years and has led to the collapse of oil production in the Apulia region. This is a significant agricultural activity, and the whole Mediterranean area is thus threatened as the olive tree is one of the predominant cultivated species in the region [95,96].

**Figure 1:**
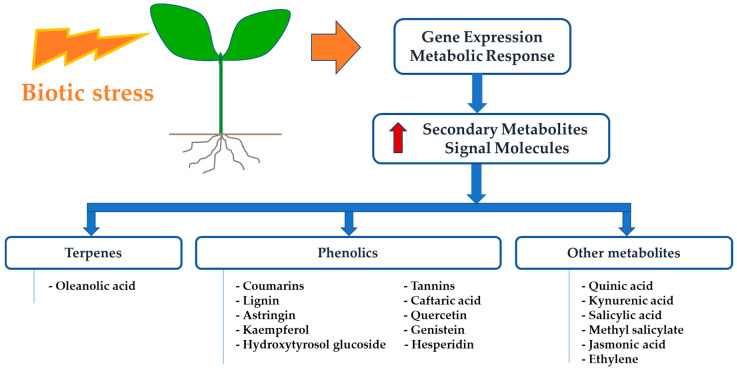
Secondary metabolites, hormones, and other compounds involved in *Xylella fastidiosa* plant interaction.

In this context, our review has addressed the main plant secondary metabolites and their potential roles in *Xf*-plant interaction. “Metabolic fingerprinting” has led to the significant acquisition of information in recent years, as it is well-suited to the discovery of chemical metabolic markers related to plant resistance [97]. However, many groups of secondary metabolites remain poorly investigated, and research into chemical host plant resistance has, for technical reasons, been limited to the identification of single compounds. However, more than one compound is typically involved in the host plant resistance process as summarized in Figure 1.

Thus, the simultaneous detection of a wide range of compounds during the pathogenesis process is necessary, and the metabolomes of resistant and susceptible plants must be directly compared to evaluate its role in disease development. This overview of the current knowledge concerning resistance against *Xf* of four principal crops has highlighted changes in the amount of some secondary metabolites (phenylpropanoid pathway correlates) as a result of bacterial infection and their roles to the limitation of pathogen titer and symptoms progression. Most research has focused on phenolic compounds produced by the host, whose bactericidal properties were frequently related to plant resistance.

In perspective, resistance breeding programs can be encouraged by new knowledge and development of metabolic engineering of crop plants. The modulation of anthocyanin pathway by the transfer of structural and regulatory genes from maize into rice increases the resistance of the transgenic plants against rice blast caused by the fungus *Magnaporthe oryzae* [98], whereas the accumulation of phenolic compounds caused by overexpression of phenylalanine ammonia lyase enhance the resistance to *Cercospora nicotinae* and *Phytophthora parasitica* pv. *nicotianae* in tobacco [99,100]. In regards to counteracting the effect of *Xf*, promising examples of genetic engineering techniques include the use of CRISPR/Cas9-mediated targeted mutagenesis of *TAS4* (Trans-Acting Small-interfering locus4) and *MYBA7* (MYeloBlastosis viral oncogene-like transcription factor) loci, involved in regulation of anthocyanin accumulation in grapevine [101]. Even if obvious PD symptoms are anthocyanin accumulation in leaves at the scorched periphery and shriveling of berries that impacts fruit quality and yield, the role of anthocyanin accumulations in grapevine tissues by *Xf* is unknown; however vector feeding preferences and olfactory cues from host anthocyanins may be important for etiologies. Besides the lack of visible pigment phenotypes in edited plants precluded pathogen challenge tests of the role of anthocyanins in host PD resistance/tolerance mechanisms, the authors demonstrate successful genome editing which can serve future characterizations of the functions of TAS4 and MYBA7 in biotic stress response pathways [101]. A further example is represented by the expression of the diffusible signal factor (DSF) molecules, a class of widely conserved quorum-sensing signals used by many Gram-negative bacterial pathogens which are also produced by *Xf*. DSF accumulates in the population of *Xf* as cell numbers increase, and their overproduction reduces its virulence, due to reduced pathogen growth and mobility within the plant [102]. Likewise, the ectopic expression of enzyme encoding DFS in transgenic tobacco and sweet orange conferred a reduction of disease symptoms and colonization of xylem vessels by *Xf* [103].

The potential for exploiting secondary plant metabolites for agricultural plant protection is huge, particularly in terms of diseases for which there is currently no available treatment, such as *Xf* diseases.
